# Toward a Politics of Care: Southeast Asian Refugee Organizing, Kinship, Care, and Reunion

**DOI:** 10.1177/15248399231164411

**Published:** 2023-03-31

**Authors:** James Huỳnh, Victoria Huỳnh, mads lê, Sheila Sy

**Affiliations:** 1 University of California, Los Angeles, CA, USA; 2 University of California, Berkeley, CA, USA

**Keywords:** care, Southeast Asian, critical refugee studies, kinship, reunion, organizing, interdependence, trauma

## Abstract

From a critical refugee studies orientation, our article redefines care within the context of myriad forms of state violence impacting Southeast Asian post-war refugee communities. Research reveals how harm is compounded at every step of Southeast Asian refugee journeys: war, forced displacement, resettlement, family separation, inherited health conditions, and generational trauma. How do we reckon with refugee trauma without conceding to it as an unchangeable fact of our lives? What knowledge might we gain by attending to the everyday work of survival in refugee communities? To answer these questions, the authors conceptualize care through (a) abolitionist organizing, (b) queer kinship and affective labor, (c) historiographic caretaking, and (d) refugee reunion.

*“care is the antidote to violence”—Saidiya* Hartman (2017)

From a critical refugee studies orientation, our article redefines care within the context of myriad forms of state violence impacting Southeast Asian post-war refugee communities. After fleeing U.S. military intervention in Southeast Asia, refugees from Cambodia, Laos, and Vietnam resettled to the United States during the vast expansion of the neoliberal and carceral state (Richie, 2012; Tang, 2015). In the 1990s, as the federal government severely reduced welfare programs, it also implemented “tough on crime” bills which poured billions of funding into state prisons and policing. Together, these policies exacerbated the criminalization of impoverished Southeast Asian refugees (Alexander & West, 2012; Tang, 2015).

Research reveals how harm has been compounded at every step of Southeast Asian refugee journeys: war, forced displacement, resettlement, family separation, inherited and/or environmental health conditions, and generational trauma (Mitchell et al., 2020). Dispersal resettlement policies in the United States were intended to reinforce separation and rewrite Southeast Asian refugees as “good” refugees who were expected to become “self-sufficient and productive members of American society” (Espiritu, 2014; Gonzalez, 2021, p. 105). However, such state-sanctioned assimilation relied on historically ableist and capitalist-driven U.S. policies. These policies were laden with rhetoric to avoid “burdening state welfare systems” and to quickly rehabilitate refugees to enter the workforce without intentional infrastructures of care and support (Gonzalez, 2021, p. 106). As evidenced by emergent ethnic enclaves and ethnoburbs, refugees resisted intentional dispersal and separation by rebuilding community networks across the United States including, but not limited to, spaces such as “Little Saigons” and “Cambodia Towns.” Yet in these community spaces, refugees often struggled with poverty, gang violence, and disproportionately high rates of incarceration for youth (Arifuku et al., 2006).

In continued resistance to such harm, we introduce our communities’ creative work of care and reconnection as public health practice. How do we reckon with refugee trauma without conceding to it as an unchangeable fact of our lives? What knowledge might we gain by attending to the *everyday work of survival* in refugee communities? To answer these questions, we offer four projects in progress that conceptualize care as (a) grassroots organizing, (b) affective labor, (c) historiographic caretaking, and (d) refugee reunion. Together, these care practices center and offer those most affected by structural violence an avenue to lead, name, and develop how to care for ourselves and our communities in our shared political contexts.

Our scholarly interventions intertwine intimate/interpersonal care to consider structural violence, building upon Public Health Critical Race Praxis principles of structural determinism, intersectionality, voice, and critical approaches (Ford & Airhihenbuwa, 2010). Furthermore, we critique medical models and health care hierarchies that relegate everyday, quotidian acts of care as undervalued or illegible, such as elderly/familial caretaking, the disability community’s “care webs,” and community mutual aid practices/projects (Piepzna-Samarasinha, 2018, p. 32). Public health tends to discuss care in terms of healthcare services mediated by the state and private entities. Our work expands the conceptualization of care to include myriad practices that take place outside of state and for-profit structures: in our homes and personal archives, in rallies and community gatherings. Grounded in feminist theory, we understand care as affective and relational, located in intimate and familial sites. We seek to understand how these family and community practices might transform notions of care in public health.

## Abolitionist Organizing as Care Intervention—Victoria Huỳnh

I present abolitionist organizing as a care intervention against Southeast Asian refugee trauma. In working in Asian Prisoner Support Committee’s (APSC) anti-deportation campaigns across California, I have built relationships with impacted refugees across prison walls and nation-state borders. Our work both challenges the criminal legal system and addresses compounded traumas in Southeast Asian refugee communities.

Refugee trauma is both a public health concern and a lived consequence of state violence. Refugees from Vietnam, Laos, and Cambodia fled U.S.-backed wars only to face structural racism, poverty, and policing upon resettlement. Policing and punitive immigration policies criminalize poverty and trauma, resulting in higher incarceration rates for young refugees. Southeast Asians are now three to four times more likely to be deported for past convictions than other immigrant communities (Mitchell et al., 2020).

In response, the movement to abolish prisons has sought to replace harmful systems of punishment with social resources that foster transformation and healing. APSC challenges carceral captivity by reconnecting incarcerated refugees to their loved ones, their histories, and their sense of self. In-prison programming is life-affirming for incarcerated individuals who have been physically, emotionally, and mentally isolated from their communities. In Restoring Our Original True Selves (ROOTS), APSC’s Ethnic Studies program in San Quentin State Prison, incarcerated refugees study and discuss the histories of war in Southeast Asia and multiple systems of oppression in the United States. They identify how trauma has shaped their lives and find belonging in a longer genealogy of resilience. Doing so begins the process of healing (Ihara, 2021).

Healing requires us to also address the material conditions responsible for refugee trauma. APSC does both: fighting criminalized deportation to rebuild community relationships. Upon their release from prison and Immigration and Customs Enforcement (ICE) detention, several formerly incarcerated refugees and immigrants in APSC have returned to their communities as advocates to free their incarcerated peers from these systems of captivity. When community members commit deeply to one another’s freedom, they challenge the logics of carceral punishment and national exclusion. When impacted refugees fight to be safe from deportation, returning home to their loved ones, they intervene against cycles of generational displacement. The open-ended promise of healing only becomes possible when our community members are freed from the systems responsible for their trauma.

In-prison and anti-deportation advocacy are key strategies to address refugee trauma. Abolitionist organizing is an urgent health intervention rooted in community knowledge rather than that of the state. When we grapple with refugee trauma as a collective issue, one which requires deep relationships and collaborative, political practice, we find richer paths toward collective well-being.

## Affective Labor of Care: Queer Vietnamese Kinship and Feminist Refugee Epistemologies—James Huỳnh

In this section, I gesture toward understandings of care rooted in queer kinship and feminist refugee epistemologies. I use ethnographic fieldwork from my time with Viet Rainbow of Orange County (VROC), a Lesbian, Gay, Bisexual, Transgender, Queer (LGBTQ) Vietnamese American community organization comprising an intergenerational membership: refugee mothers, queer and trans U.S.-born youth, and older queer and trans immigrants and refugees.

I argue that the refugee mothers in VROC should be viewed as knowledge producers and practitioners of queer kinship and care in ways that subvert conventional practices of family and kinship, and racialized motherhood. Within the racial and heterosexist structure of the United States, queer and trans Vietnamese children and adults along with their refugee mothers “need all the help they can get if they are going to survive this world” (Chambers-Letson, 2018, loc. 1987). Through their affective care labor, I demonstrate that the mothers engage in a praxis aligned with feminist refugee epistemologies, which underscore the “practices of life making. . . the hidden and overt injuries but also the joy and survival practices that play out in the domain of the everyday” (Espiritu & Duong, 2018, p. 588).

Caring labor is entirely immersed in the corporeal. Thus, affective labor can be understood from feminist analyses of “women’s work” as “labor in the bodily mode” (Hardt, 1999, p. 6). I use affective labor to describe the different kinds of bodily care work that VROC mothers do for each other, their chosen kin, and their community organizing efforts. From cooking food together, teaching and learning from each other about queerness, and providing one another emotional and physical social support, the mothers’ affective labor creates a sense of community and belonging.

In this work, the moms often return to the phrase, “gia đình là gia đình [family is family].” This continuous signification of the family illustrates the mothers’ deployment of its associated affective relationships of care, labor, and belonging. Through their *care*ful work of connecting to other Vietnamese parents, eating meals in their homes, and sharing stories, these are the sorts of affective care labor that the moms do to expand their community’s understanding of queerness and family.

Important to public health, this understanding of care destabilizes the popular House model of social support, which separates emotional and instrumental support (House, 1981). Queer notions of chosen family tend to reject blood kinship for non-biological relationships. Yet, affective labor rooted in queer feminist refugee epistemologies of care helps reimagine chosen family as a practice of reconciling with our blood-related family members, rather than simply forsaking them.

## Chronic Possibilities and Historiographic Caretaking: Vietnamese Refugee Families as Sites of Care and Refusal—mads lê

I propose that the refugee family can operate in ways that supersede capitalist medical systems that structure our everyday living. To ground my intervention, I interrogate structures of medical encounters via my family’s personal archival documents. From my maternal family’s early medical records from the Naval Air Station at Cubi Point, or the Subic Bay Refugee Camp in the Philippines, alongside autoethnographic notes from various caretaking periods in my life, I trace the contours of my family’s relationship to medical infrastructures, health care encounters, and complexities of caretaking/giving. These materials lend insight into the White American body politic as a structure of/for ableism that implicates our choices to care for and/or to determine the livelihoods of our loved ones.

In the Visa medical examination, examiners assess “DANGEROUS CONTAGIOUS DISEASES,” “MENTAL CONDITIONS,” “Physical defect, disease, or disability serious in degree or permanent in nature amounting to a substantial departure from normal physical well-being,” and “Minor Conditions.” This document scaffolds our understanding of ableist medical processes: the form itself administratively polices bodies to “fit” within the American “ideal” of able-bodiedness at the site of the refugee camp prior to reaching American soil (Dolmage, 2011, p. 58). Tracing these histories helps us bridge the relationship between the creation of a healthy, white national body politic to the individual racialized bodies that are always already understood as other, therefore, contaminated, diseased, and contagious.

Resurfacing these documents 10 years after my maternal aunt’s death felt surreal because she was the first family member to depart and index for how important it was to understand our medical histories as *chronic possibilities*. When reflecting with my mother, we reminisced on how she felt when she first learned about my aunt Trúc’s lupus. My mother shares that lupus could have been “dormant” until externally triggered. Dormancy, to “nằm nghĩ [to lay to rest],” tethers chronic possibility in relation to broader contexts of diagnoses, illnesses, care-seeking, history, and Eastern medicinal principles (Tu, 2021, p. 36). Even though my mother insisted my aunt heed “official” medical advice to not have a child—when aunt Trúc decided to have a baby—my mother left to support her birthing process, do the aftercare, and cook meals. Aunt Trúc’s refusal indexes her autonomy in facilitating the care she wanted rather than adhering to what medical systems dictated for her.

By centering my family’s encounters with the United States visa medical exemption process, their caregiving relationships, and my autoethnographic experiences, I gesture toward imaginative possibilities and grounded practices in what I call *historiographic caretaking* as a means to respond to the chronic possibilities of our environmental and historical inheritances. Historiographic caretaking is the process of documenting our care practices while contextualizing that environments, traumas, and histories are not single events but a series of chronic possibilities embedded in our lineages. Critical to consider in relation to public health scholarship and existing state-sanctioned medical models of care, historiographic caretaking provides a theoretical praxis to both document and index acts of refusal and familial caretaking as experiments in anti-capitalist practice and disability justice.

## Refugee Reunion: Directions for Cambodian Refugee Intergenerational Healing—Sheila Sy

As part of the largest group to be resettled in the United States nearly four decades ago, deportation of refugees back to Cambodia compounds existing refugee trauma across generations. Focusing on Cambodian refugees’ war-based displacement, critical refugee studies scholar Khatharya Um interrogates healing through diasporic return. She suggests that return “is a step toward recovery of the past and of the self and hopefully of wholeness” (Um, 2015, p. 252). However, the forced return of refugees to Cambodia presents contradictory notions of home and homeland, restructuring Cambodian refugee diasporas. This brings me to ask, how must we expand our understanding of return to fully reckon with the harms and possibilities for healing within the context of deportation? My project draws from critical refugee studies and feminist geography and utilizes ethnography to conceptualize reunion as a site of healing from generational displacement.

Reunion necessarily expands upon Um’s concept of return in that reunion is geographically flexible. To define reunion, I first interrogate reunion as a spatial concept. A feminist understanding of space calls out the failures of the spatial imagination which assume space is merely a surface to be traveled across and conquered (Massey, 1994). Both reunion and return reflect Massey’s relational approach to understanding space. Understanding these concepts as a product of interrelations gives way to a radical openness of the future and political possibilities. Reunion is a site of possibility. Although reunion does not guarantee healing, like return, reunion is a step toward healing. Thinking spatially provides the foundation to understand reunion as a directional marker for the remaking of refuge relations and personhood.

Although refugee deportation reinscribes past displacement and trauma, refugee deportation also engenders a new politic and possibilities for healing. Reunion of refugees with family, community, land, and self is foregrounded by the life-affirming experiences of resisting transnational state violence. Understanding the Cambodian refugee through the lens of reunion provides a potent counterweight to the construction of the “deportable” refugee in that reunion expands the conversation beyond the horrors of war and genocide. Refugee reunion re-centers the experiences of Cambodian refugees to make visible the profound loss, as well as the resistance and joys of those directly affected.

The Southeast Asian refugee political movement stands at the precipice of political success. In recent years, the movement has seen wins including returns of a handful of individual deportees from Cambodia, countless prevention of deportations, and the introduction of landmark legislation aimed at ending Southeast Asian refugee deportation completely. Win or lose policy change, however, refugee reunion underscores that what is at stake extends beyond the political. Alternatively, what is at stake is healing generational trauma. For public health practitioners, refugee reunion presents a theoretical framework to reconsider the ways refugees’ past structures the possibilities for healing in the present.

## Implications for Practice

In this article, we foreground a collective approach to public health. Rather than engaging with trauma as an individualized or pathological problem, we study Southeast Asian refugee trauma as a lived consequence of compounded structural violence. We offer the following implications for public health practitioners and researchers:

1. Public health practitioners must work alongside abolitionist organizers to up-end the carceral systems responsible for poor health outcomes and generational trauma.
2. The *affective labo*r of Vietnamese mothers and the queer kinship relationships built from that labor form a care practice that provides vital social support to LGBTQ youth.
3. Health and illness cannot be anchored in singular events but rather comprises a series of factors including war environments, refuge, immigration, and resettlement (*chronic possibility)*. To address this, documenting refugee care practices and remembering through collective mourning can be forms of healing (*historiographic caretaking*).
4. *Refugee reunion* can be both an intimate site of repair within family separation and an opening for new social movements, grounded in shared histories.

Taken together, rather than simply resolving refugee trauma, which is often framed as irrecoverable loss, our article puts forth a transformative politics of care that exists outside of recuperation. Drawing on critical refugee studies, we urge public health practitioners and researchers to center Southeast Asian refugees’ ways of knowing, being, and caring as sites of intervention.
